# Prediction of cardiac arrest in critically ill patients presenting to the emergency department using a machine learning score incorporating heart rate variability compared with the modified early warning score

**DOI:** 10.1186/cc11396

**Published:** 2012-06-21

**Authors:** Marcus Eng Hock Ong, Christina Hui Lee Ng, Ken Goh, Nan Liu, Zhi Xiong Koh, Nur Shahidah, Tong Tong Zhang, Stephanie Fook-Chong, Zhiping Lin

**Affiliations:** 1 Department of Emergency Medicine, Singapore General Hospital, Outram Road, Singapore 169608, Singapore; 2 Yong Loo Lin School of Medicine, National University of Singapore, 12 Medical Drive, Singapore 117598, Singapore; 3 Duke-NUS Graduate Medical School, 8 College Road, Singapore 169857, Singapore; 4 Department of Clinical Research, Singapore General Hospital, Outram Road, Singapore 169608, Singapore; 5 School of Electrical and Electronic Engineering, Nanyang Technological University, 50 Nanyang Avenue, Singapore 639798, Singapore

## Abstract

**Introduction:**

A key aim of triage is to identify those with high risk of cardiac arrest, as they require intensive monitoring, resuscitation facilities, and early intervention. We aim to validate a novel machine learning (ML) score incorporating heart rate variability (HRV) for triage of critically ill patients presenting to the emergency department by comparing the area under the curve, sensitivity and specificity with the modified early warning score (MEWS).

**Methods:**

We conducted a prospective observational study of critically ill patients (Patient Acuity Category Scale 1 and 2) in an emergency department of a tertiary hospital. At presentation, HRV parameters generated from a 5-minute electrocardiogram recording are incorporated with age and vital signs to generate the ML score for each patient. The patients are then followed up for outcomes of cardiac arrest or death.

**Results:**

From June 2006 to June 2008 we enrolled 925 patients. The area under the receiver operating characteristic curve (AUROC) for ML scores in predicting cardiac arrest within 72 hours is 0.781, compared with 0.680 for MEWS (difference in AUROC: 0.101, 95% confidence interval: 0.006 to 0.197). As for in-hospital death, the area under the curve for ML score is 0.741, compared with 0.693 for MEWS (difference in AUROC: 0.048, 95% confidence interval: -0.023 to 0.119). A cutoff ML score ≥ 60 predicted cardiac arrest with a sensitivity of 84.1%, specificity of 72.3% and negative predictive value of 98.8%. A cutoff MEWS ≥ 3 predicted cardiac arrest with a sensitivity of 74.4%, specificity of 54.2% and negative predictive value of 97.8%.

**Conclusion:**

We found ML scores to be more accurate than the MEWS in predicting cardiac arrest within 72 hours. There is potential to develop bedside devices for risk stratification based on cardiac arrest prediction.

## Introduction

In the emergency department (ED), triage is used to assess the severity of patients' conditions and to assign appropriate treatment priorities. This clinical process entails the rapid screening of large numbers of patients to assess severity and assign treatment. Risk stratification for cardiac arrest and other adverse cardiac outcomes plays an essential role in the management of chest pain patients in the ED [1]. Medical decisions for disposition as well as the required level of intensive monitoring rest on this perceived risk [2]. Risk stratification is a necessity because medical resources are never sufficient for all patients to be attended instantaneously in busy EDs and hospitals, with limited numbers of doctors, nurses, monitored beds, resuscitation facilities, intensive care beds, operating theaters, and so forth. Quick identification of patients of higher severity, who would more urgently need and potentially benefit from such resources, is thus important.

Current risk-stratification systems are based on clinical judgment and traditional vital signs including heart rate, respiratory rate, blood pressure, temperature, and pulse oximetry [3]. Unfortunately vital signs have not been shown to correlate well with short-term or long-term clinical outcomes [4]. The modified early warning score (MEWS) is one such widely used tool (Table 1). The MEWS is based on physiological parameters: systolic blood pressure, pulse rate, respiratory rate, temperature and AVPU score (A for 'alert', V for 'reacting to vocal stimuli', P for 'reacting to pain', U for 'unconscious'). We selected the MEWS as our comparator tool because it is widely used in the UK and in Commonwealth countries to identify patients at risk of deterioration, and raised MEWS values are associated with increased mortality [5]. The MEWS can be relatively quickly calculated during triage, without the need for laboratory test results, for example. Other studies carried out in the UK have shown good results in predicting poor outcomes in their patient groups [5-7]. However, the assessment of the AVPU score is a relatively subjective element in the scoring. Also, the range of sensitivities and specificities are dependent on the cutoff score used and the MEWS requires some training to be accurate.

**Table 1:** Modified early warning score

Score	Respiratory rate (breaths/minute)	Heart rate (beats/minute)	Systolic blood pressure (mmHg)	Temperature (°C)	AVPU
3	-	-	≤ 70	-	-
2	≤ 8	≤ 40	71 to 80	≤ 35	-
1	-	41 to 50	81 to 100	35.1 to 36	-
0	9 to 14	51 to 100	101 to 199	36.1 to 38	Alert
1	15 to 20	101 to 110	-	38.1 to 38.5	Reacting to voice
2	21 to 29	111 to 129	≥ 200	≥ 38.6	Reacting to pain
3	> 29	> 129	-	-	Unresponsive

AVPU, A for 'alert', V for 'reacting to vocal stimuli', P for 'reacting to pain', U for 'unconscious'.

Heart rate variability (HRV) is a non-invasive measurement for investigating autonomic influence on the cardiovascular system that has generated significant interest in recent scientific literature [8]. HRV may be defined as the change in the time interval between heartbeats, from beat to beat. HRV is controlled by the autonomic nervous system, including the sympathetic nervous system and the parasympathetic nervous system [9,10]. There is recognition of a significant relationship between the autonomic nervous system and cardiovascular mortality, including sudden cardiac death [11,12]. Recent studies have found strong associations between HRV from short-term (2 to 30 minutes) electrocardiogram (ECG) recordings and post-acute myocardial infarction mortality [13,14]. These associations suggest that short-term HRV measurements may serve as a rapid risk-stratification tool for adverse cardiac events.

Machine learning (ML) is based on the way the human brain approaches pattern recognition tasks, providing an artificial intelligence-based approach to solve classification problems. A model is learned during the training process using previously known input-output pairs. The trained model is then tested with new data. Various ML topologies are available, including single-layer and multi-layer feedforward networks. ML adjusts weights of hidden layers during training to minimize an error function [15].

In this study, we aim to validate a novel ML score incorporating HRV for risk stratification of critically ill patients presenting to the ED by comparing the area under the curve, sensitivity and specificity for prediction of cardiac arrest with the MEWS.

## Materials and methods

### Study design

We conducted a prospective, nonrandomized, observational cohort study, looking at critically ill patients attended by the Singapore General Hospital ED. Singapore General Hospital is the oldest and largest acute tertiary hospital in Singapore. The hospital accounts for about one-third of all acute-care public-sector beds and about one-quarter of acute beds nationwide. It is a Level 1 Trauma Centre for Singapore. Annually, about 60,000 patients are admitted to its wards and another 600,000 patients are attended to in its Specialist Outpatient Clinics. The ED sees between 300 and 500 patients a day.

All public hospitals in Singapore use a national Patient Acuity Category Scale (PACS) for triage at the ED. PAC 1 patients are the most critically ill and would therefore be required to be attended to without delay. They would most probably require maximum allocation of staff and equipment resources for initial management. The severity of their symptoms requires very early attention, failing which early deterioration of their medical status is likely. PAC 2 patients are nonambulant and would appear to be in a stable state on initial cardiovascular examination and are not in danger of imminent collapse. PACS 3 patients are ambulant and PACS 4 patients are nonemergencies. PACS is a symptom-based triage system and does not have strict physiological criteria (for example, vital sign cutoff values).

At Singapore General Hospital ED, all patients are initially triaged by a nurse, and those with airway, breathing and circulation problems, or those thought to be possibly unstable and needing close monitoring, are routinely put on ECG monitoring using the LIFEPAK^® ^12 defibrillator/monitor (Physio-Control, Redmond, WA, USA). These would be PACS 1 patients and some PACS 2 patients.

### Patient recruitment and eligibility

All patients older than 18 years of age requiring continuous ECG monitoring with PACS 1 or PACS 2 were eligible. Patients in nonsinus rhythm (asystole, supraventricular arrhythmias, ventricular arrhythmias, complete heart block, and pacemaker rhythm) were excluded because HRV metrics are not reliable for nonsinus rhythms. Patients who were subsequently discharged against medical advice or transferred to another hospital for care were considered lost to follow-up and excluded from the study as clinical outcomes could not be determined. We also excluded cases with a high percentage of artifacts, nonsinus beats, and ectopics combined together (> 30% of recorded tracing); cases with ≤ 30% of artifacts and so forth were included, but the nonsinus segments of the tracings were trimmed off. Patients were only recruited during office hours. The initial set of vital signs and HRV parameters obtained during triage was recorded for this study. HRV recordings ranged from 5 to 30 minutes. Ethics approval was obtained from the Singhealth Centralised Institutional Review Board (CIRB Approval No. 2006/018/C) with a waiver of patient consent for the study. Patients were recruited from June 2006 to June 2008.

### Hospital outcomes

The primary outcome was cardiac arrest within 72 hours of presentation to the ED. The event of cardiac arrest was defined as sudden unexpected death or a resuscitation event requiring cardiopulmonary assistance (chest compressions and/or defibrillation). This assistance was thought to probably reflect a primary cardiac event/etiology. Information regarding the nature of death or the resuscitation event was extracted from clinical notes. The secondary outcome was death after admission (in-hospital death during current admission, including within 72 hours). This endpoint would have included patients dying from primary cardiac as well as noncardiac etiologies. Patients were followed up until discharge or in-hospital death. Information regarding the nature of death was extracted from clinical notes. For patients who were discharged before 72 hours, electronic medical records providing information on admission to all public hospitals in Singapore were reviewed for study outcomes.

### Data collection and processing

ECG tracings (long lead I, II, III and 12-lead ECG data) obtained during initial presentation from critically ill patients on a LIFEPAK 12 defibrillator/monitor were downloaded using the CODE-STAT Suite data review software (version 5.0; Physio-control). Lead II ECGs sampled at 125 Hz were extracted as text files for HRV analysis using CODE-STAT™ and proprietary ECG extraction software (Physio-Control); 125 Hz is the sampling rate used by the defibrillator monitor. Since we are primarily looking at the QRS complexes and not interested in high-frequency features of the ECG, this is a sufficient rate of digitization. Cases with ECG recordings were prospectively identified and had identity confirmed by querying ED charts and records. A minimum ECG recording of 5 minutes is required in order to accurately calculate HRV metrics.

**Figure 1:**
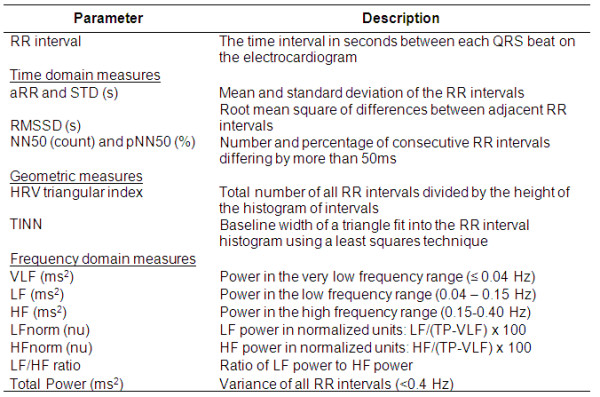
**List of heart rate variability parameters**.

The ECG records were converted into text (ASCII) files using an extraction program available with CODE-STAT. The processing program was embedded with a MATLAB code (R2009a; The Mathworks, Natick, Massachusetts, USA), which was used to process the ECG signals to obtain the HRV variables (see Figure 1), in accordance with the guidelines outlined by the Taskforce of the European Society of Cardiology [16]. The raw ECG data were first preprocessed to reduce the effects of noise and artifacts using a 5 to 28 Hz band-pass filter. This frequency range has been found to enhance the QRS complex against the background noise for easier peak detection [17]. A modified threshold-plus-derivative method was used to detect the QRS complexes, and all ectopics and other nonsinus beats were excluded in accordance with the guidelines outlined by the Taskforce of the European Society of Cardiology [16], using an automatic detection algorithm. RR intervals were then calculated based on the sinus rhythm. The beat detection and labeling techniques have previously been validated against manually annotated data from the MIT-BIH database [18] and have been found to perform with high accuracy [19]. Ectopic beats were identified by the size and shape of the QRS complexes as well as the distances between successive beats. The height of the QRS complex, width, and RR interval were also considered. In addition, atrial fibrillation was identified manually by study engineers during retrospective verification of ECGs.

The ECG tracings were then analyzed for heart rate variability, with both time-domain and frequency-domain analyses. Other variables collected were age, gender, medical history including ischemic heart disease, diabetes mellitus and chronic renal failure, heart rate, blood pressure, respiratory rate, Glasgow Coma Scale (GCS), etiology, and medication history. Vital signs (heart rate, blood pressure, and oxygen saturation (SpO_2_)) were measured using the Propaq CS Monitor (Welch Allyn, Skaneateles Falls, NY, USA) vital signs monitor in the ED. The GCS and respiratory rate were recorded at the time of vital sign measurement. AVPU scores were recorded at triage. Tympanic temperatures of the patients were taken using a tympanic thermometer. AVPU scores were scored according to the best response during data collection. The collected data were used to calculate a MEWS for each patient recruited.

HRV variables measured included time-domain, frequency-domain, and geometric parameters. The frequency-domain parameters were calculated based on estimates of power spectral density, obtained using the Lomb-Scargle periodogram that is commonly used for unevenly sampled sequences. Use of the Lomb-Scargle periodogram eliminates the need for interpolation or resampling of the sequences [20,21].

### Machine learning score prediction

A ML-based prediction model - utilizing age, HRV parameters, and vital signs - was proposed to compute risk score on patient's hospital outcome [22]. This model was run on a MATLAB code (R2009a; The Mathworks). In contrast to traditional mathematical logistic regression approaches, this is a multivariate, nonparametric, black-box approach. This approach overcomes problems faced by traditional statistical models of colinearity and overfitting. Assuming that each patient's data can be represented as a vector of HRV parameters and vital signs, the scoring system is built based on the calculation of geometric distances among a set of feature vectors (that is, multiple patients). Classifier selection plays a key role in building an efficient prediction system. In this study, the support vector machine was adopted to map feature vectors onto a higher dimensional space and find an optimal pattern-separating hyperplane [23,24]. The support vector machine has shown satisfactory performances in many areas including ECG beat classification [25], EEG analysis [26], and text classification [27].

The calculation of the ML score is straightforward. First, cluster centers of both positive and negative samples are calculated in Euclidean space, where positive samples are patients with cardiac arrest or death as outcomes and negative samples are patients without the above outcomes. A score is then computed by evaluating Euclidean distances between a patient's data and both cluster centers. Last, the risk score is fine-tuned through a novel imbalanced learning strategy. If the predicted outcome is positive, the risk score will be increased.

As shown in Table 2 the database consists of a majority group of normal samples and a minority group of samples with abnormal outcomes (cardiac arrest or death); that is, the dataset is imbalanced. Common ML algorithms cannot be directly implemented on this imbalanced database for score tuning, because the majority class will dominate the learning process and leads to poor generalization performance on new patients from the minority class. The solution to handling data imbalance is to create a decision ensemble [28]. Our method partitions the samples of majority class into *N *nonoverlapped groups, with each group joined by minority samples. By doing so, *N *balanced datasets are created, on which a prediction model is trained to distinguish minority and majority classes. The ML algorithm was trained and validated using a leave-out-one strategy.

**Table 2:** Baseline characteristics of study patients

Characteristic	No cardiac arrest within 72 hours (*n *= 882)	Cardiac arrest within 72 hours (*n *= 43)	*P *value^a^
Age (years)			
Median (IQR)	62 (50 to 74)	70 (59 to 78)	0.018
Male gender	542 (61.5)	31 (72.1)	0.198
Race			
Chinese	586 (66.4)	35 (81.4)	
Malay	130 (14.7)	5 (11.6)	0.134
Indian	118 (13.4)	1 (2.3)	
Others	48 (5.4)	2 (4.7)	
Diagnosis grouping^b^			
Cardiovascular	359 (40.7)	9 (20.9)	
Respiratory	137 (15.5)	10 (23.3)	
Neurological	87 (9.9)	4 (9.3)	
Gastrointestinal	46 (5.2)	0 (0)	
Renal	25 (2.8)	0 (0)	
Endocrine	58 (6.6)	4 (9.3)	0.275
Infectious diseases	59 (6.7)	1 (2.3)	
Vascular	22 (2.5)	3 (7.0)	
Trauma	31 (3.5)	2 (4.7)	
Cancer	28 (3.2)	9 (20.9)	
Others	30 (3.4)	1 (2.3)	
Medical history^c^			
Diabetes	295 (33.4)	5 (11.6)	0.405
Hypertension	472 (53.5)	18 (41.9)	0.876
Heart disease	292 (33.1)	12 (27.9)	0.868
Renal disease	115 (13.0)	6 (14.0)	0.251
Respiratory disease	103 (11.7)	7 (16.3)	0.222
Stroke	63 (7.1)	0 (0)	1.000
Cancer	69 (7.8)	2 (4.7)	1.000
Others	523 (59.3)	32 (74.4)	0.204
Prior medical therapy^d^			
Beta-blockers	227 (25.7)	14 (32.6)	0.726
Calcium-channel blockers	165 (18.7)	11 (25.6)	0.432
Digoxin	36 (4.1)	2 (4.7)	0.415
Amiodarone	12 (1.4)	0 (0)	1.000
Other anti-arrythmics	5 (1.4)	0 (0)	1.000

Data shown are numbers (%) unless otherwise stated. IQR, interquartile range (25th to 75th percentiles). ^a^*P *value from either the chi-square test or the Mann-Whitney test as appropriate. ^b^Based on admitting emergency physician clinical diagnosis. ^c^Medical history at presentation to the emergency department. ^d^Prior outpatient medical therapy at presentation to the emergency department.

### Statistical analyses

Continuous variables are presented as means (standard deviation) or medians (interquartile range) and were analyzed using a two-tailed Student's *t *test and the Wilcoxon rank-sum test, respectively. Categorical variables are presented as numbers (percentage) and were analyzed using the chi-square test or Fischer's exact test when appropriate.

The ML score and the MEWS were calculated for all patients and analyzed for a significant association between the scores and the incidence of cardiac arrest or death, and adverse cardiac events. Receiver operating characteristic (ROC) curves were based on the continuous measurements of the ML score and the ordinal measurements of the MEWS.

Patients were categorized into low, intermediate, and high risk groups according to their ML scores, based on review of the data (selecting the cutoff values that provided the best discrimination): low risk, ML score 0 to 40; intermediate risk, ML score 41 to 60; and high risk, ML score 61 to 100.

The area under the receiver operating characteristic curve (AUROC) for the ML score and the MEWS was calculated and compared using a *z*-statistic made from the difference between the two AUROCs divided by the standard error of the difference in the AUROCs [29]. The confidence interval (CI) for reporting the difference between the two AUROCs was also derived. Statistical comparison tests between the ML score and the MEWS for sensitivity and specificity were done by applying the McNemar test to the disease group (comparison of sensitivities) and the nondiseased group (comparison of specificities) [30]. Similarly, statistical comparison tests between ML and the MEWS for the predictive values were done using the above same method [30], whereby the test statistics had a chi-square distribution with one degree of freedom. A statistical comparison test for the likelihood ratio of a positive test was not performed, however, because no well-established method of comparison was found in the literature. Whenever possible, the 95% CI for the difference in diagnostic value between the two scoring methods was provided. For the predictive values, the CI of the difference was not readily computable by well-established methods, but significance tests were carried out for these comparisons. Moreover, separate CIs of the predictive value within each scoring method are presented. A general rule of thumb is that CIs can overlap as much as 29% and the statistics can still be significantly different (see [31] chapter 2.6: overlapping confidence intervals do not imply nonsignificance). The ML score was further categorized into low, intermediate, and high risk scores and was tested for a significant relationship with the rates of cardiac arrest or death. Optimum cutoff points were determined using sensitivity and specificity analysis.

Unless otherwise specified, *P *< 0.05 was considered to indicate statistical significance. All data were stored with Excel (Microsoft Office 2007; Microsoft, Redmond, WA, USA) and imported into SPSS software (version 17.0; SPSS Inc., Chicago, IL, USA) and STATA software (version 11.1; STATA Corporation, College Station, TX, USA) for statistical analysis.

## Results

### Baseline characteristics

A total of 1,025 ECG tracings were collected during this period. Out of these tracings, 100 were excluded due to a high percentage of artifacts, nonsinus beats, ectopics, or missing data. A total of 925 patients were recruited during the study. Table 2 shows the characteristics of the study patients. The diagnosis grouping shown was based on the admitting emergency physician clinical diagnosis. The largest diagnosis grouping is the cardiovascular group at 468 (50.6%), followed by the respiratory group at 147 (15.9%).

### Outcomes

Forty-three (4.6%) of the total sample developed cardiac arrest within 72 hours, while 86 (9.3%) died after admission (including those deaths within 72 hours). The respiratory diagnosis group had the largest number of primary outcomes at 10 (23.3%), followed by cardiovascular and cancer groups both at nine incidences (20.9%). Both the gastrointestinal and renal groups did not have any patients with primary outcome (cardiac arrest). The respiratory diagnosis group has the largest number of secondary outcomes at 19 (22.1%), followed by the cardiovascular diagnosis group at 18 (20.9%).

Table 3 shows the relationship of the predictor factors with the outcome of cardiac arrest within 72 hours and death after admission. Those factors found to have significant association (*P *< 0.05) with the primary outcome included the GCS, pulse rate, respiratory rate, SpO_2_, aRR, avHR, sdHR, RR triangular index, LS-VLF power, LS-HF power, LS-LF norm, LS-HF norm, and MEWS. Those factors found to have significant association (*P *< 0.05) with the secondary outcome included the GCS, respiratory rate, SpO_2_, aRR, avHR, RMSDD, RR triangular index, TINN, LS-VLF power, LS-HF power, LS-LF norm, LS-HF norm, LF/HF ratio, and MEWS.

**Table 3:** Measurements of MEWS, vital signs and heart rate variability of study patients

Variable	No cardiac arrest within 72 hours (*n *= 882)	Cardiac arrest within 72 hours (*n *= 43)	*P *value^a^	No death (*n *= 839)	Death (*n *= 86)	*P *value^a^
Age	61 (16)	66 (16)	0.047	61 (16)	69 (16)	< 0.001
Vital signs						
Glasgow Coma Scale	15 (15 to 15)	15 (11 to 15)	0.002	15 (15 to 15)	15 (10 to 15)	< 0.001
Temperature (°C)	37 (1)	37 (1)	0.118	37 (1)	37 (1)	0.280
Pulse rate (beats/minute)	96 (30)	106 (25)	0.026	96 (30)	102 (25)	0.055
Respiratory rate (breaths/minute)	19 (5)	20 (5)	0.040	19 (5)	20 (5)	0.021
Systolic BP (mmHg)	136 (38)	125 (34)	0.082	136 (37.899)	130 (40.761)	0.207
Diastolic BP (mmHg)	77 (22)	75 (20)	0.460	78 (21)	73 (23)	0.076
Oxygen saturation (%)	96 (6)	93 (13)	< 0.001	96 (6)	94 (9)	0.001
Pain score	0 (0 to 3)	0 (0 to 3)	0.825	0 (0 to 3)	0 (0 to 0)	0.010
HRV variables						
aRR (s)	0.718 (0.177)	0.621 (0.149)	< 0.001	0.721 (0.175)	0.644 (0.176)	< 0.001
STD (s)	0.053 (0.033)	0.057 (0.047)	0.490	0.054 (0.033)	0.051 (0.044)	0.534
avHR (beats/minute)	89.004 (20.566)	102.692 (22.136)	< 0.001	88.582 (20.317)	99.971 (22.975)	< 0.001
sdHR (beats/minute)	6.463 (3.740)	8.152 (5.531)	0.005	6.473 (3.731)	7.204 (4.877)	0.094
RMSSD (ms)	0.039 (0.041)	0.048 (0.054)	0.272	0.038 (0.039)	0.048 (0.063)	0.184
nn50 (count)	571 (1,268)	484 (748)	0.655	547 (1,178)	765 (1,800)	0.123
pnn50	7.048 (12.268)	6.561 (9.667)	0.797	6.878 (11.773)	8.460 (15.411)	0.251
RR triangular index	2.475 (1.000)	2.046 (0.736)	0.006	2.486 (1.000)	2.158 (0.840)	0.004
TINN (ms)	0.217 (0.130)	0.189 (0.148)	0.170	0.219 (0.130)	0.184(0.142)	0.017
VLF power (ms^2^)	0.131 (0.102)	0.099 (0.084)	0.045	0.133 (0.101)	0.098 (0.097)	0.002
LF power (ms^2^)	0.057 (0.042)	0.056 (0.045)	0.876	0.058 (0.043)	0.053 (0.040)	0.319
HF power (ms^2^)	0.080 (0.070)	0.103 (0.083)	0.032	0.079 (0.070)	0.100(0.077)	0.008
Total power (ms^2^)	0.268 (0.135)	0.259 (0.131)	0.673	0.269 (0.135)	0.251 (0.137)	0.223
LF power (nu)	45.430 (18.489)	36.459 (15.798)	0.002	45.938 (18.586)	35.990 (14.452)	< 0.001
HF power (nu)	54.566 (18.487)	63.541 (15.798)	0.002	54.058 (18.583)	64.010 (14.452)	< 0.001
LF/HF ratio	1.205 (1.284)	0.802 (1.171)	0.043	1.232 (1.299)	0.742 (0.992)	0.001
MEWS	2 (1 to 4)	4 (2 to 5)	< 0.001	2 (1 to 4)	4 (2 to 5)	< 0.001

Data shown are mean (standard deviation) or median (interquartile range, 25th to 75th percentiles). BP, blood pressure; HRV, heart rate variability; MEWS, modified early warning score. ^a^*P *value from either an unpaired *t *test or the Mann-Whitney test as appropriate.

**Figure 2:**
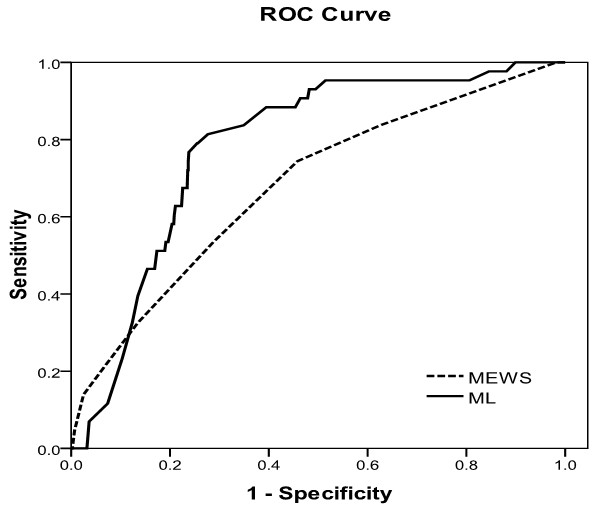
**Machine learning score and modified early warning score predicting cardiac arrest within 72 hours**. Receiver operating characteristics (ROC) curve analysis of the machine learning (ML) score and the modified early warning score (MEWS) in predicting cardiac arrest within 72 hours.

**Figure 3:**
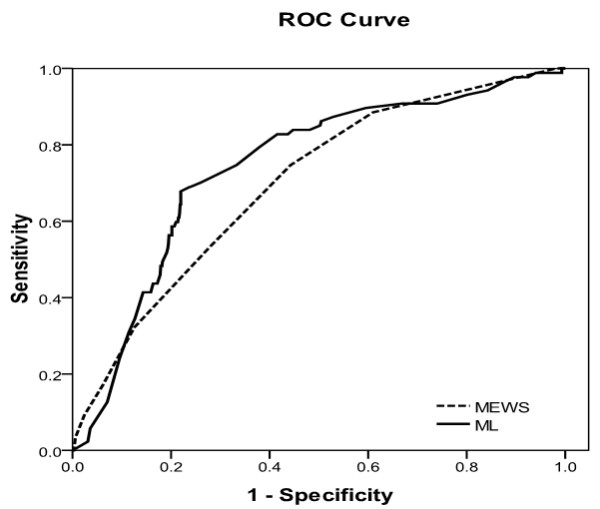
**Machine learning score and modified early warning score predicting death within 72 hours**. Receiver operating characteristics (ROC) curve analysis of machine learning (ML) score and the modified early warning score (MEWS) in predicting death after admission.

The ROC and AUCs of the ML score and the MEWS for predicting cardiac arrest within 72 hours or death after admission are illustrated in Figure 2 and 3, respectively.

**Figure 4:**
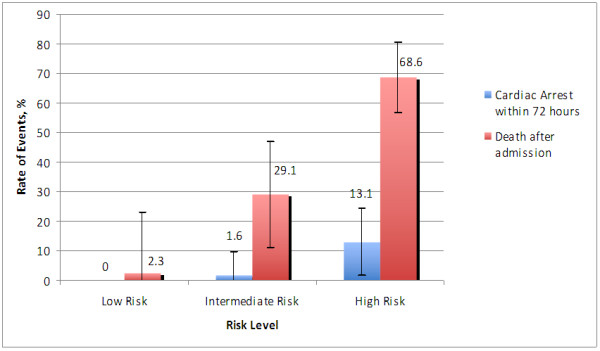
Rates of cardiac arrest within 72 hours and death in relation to machine learning score. *, **.

Eighty-nine patients (9.6%), 576 patients (62.3%), and 260 patients (28.1%) were in the low, intermediate, and high risk ML score groups, respectively. Rates of cardiac arrest within 72 hours were 0%, 1.6% (95% CI: 6.59 to 9.79), and 13.1% (95% CI: 1.75 to 24.45) in the low, intermediate, and high risk groups, respectively, as shown in Figure 4. Rates of death after admission were 2.3% (95% CI: 18.48 to 23.08), 29.1% (95% CI: 11.30 to 46.90), and 68.6% (95% CI: 56.76 to 80.44) in the low, intermediate, and high risk groups, respectively.

Table 4 shows the sensitivity, specificity, positive predictive values, and negative predictive values for the ML score and the MEWS for predicting cardiac arrest within 72 hours or death after admission. The AUROC of the ML score was higher compared with the MEWS for the primary outcome of cardiac arrest (0.781 vs. 0.680, difference in AUROC: 0.101, 95% CI: 0.006 to 0.197; *P *= 0.037) but not for the secondary outcome of death. For prediction of cardiac arrest within 72 hours after presentation, the sensitivity and specificity of the ML score were 81.4 and 72.3, respectively, compared with sensitivity and specificity of the MEWS being 74.4 and 54.2, respectively (difference in sensitivity: 7.0, 95% CI: -11.1 to 21.9; and difference in specificity: 18.1, 95% CI: 14.3 to 22.0). Specificity for cardiac arrest but not sensitivity was thus significantly higher in ML compared with the MEWS. The positive predictive value of the ML score was higher (12.5, 95% CI: 9.0 to 17.1) compared with the positive predictive value of the MEWS (7.4, 95% CI: 5.3 to 10.3; *P *< 0.001). The likelihood ratio of a positive test for the ML score was higher (2.94, 95% CI: 2.46 to 3.52) compared with the likelihood ratio of the MEWS (1.62, 95% CI: 1.34 to 1.96). As for prediction of death after admission, the specificity of the ML score (73.9) was higher compared with the specificity of the MEWS (55.7) (difference in specificity: 18.2; 95% CI: 14.3 to 22.2). The positive predictive value for the ML score (21.5, 95% CI: 16.9 to 26.9) was higher compared with the positive predictive value of the MEWS (14.7, 95% CI: 11.5 to 18.4; *P *< 0.001). The likelihood ratio of the ML score (2.67, 95% CI: 2.23 to 3.20) was higher compared with the likelihood ratio of the MEWS at (1.68, 95% CI: 1.45 to 1.94).

**Table 4:** Discriminatory values of the machine learning score and the modified early warning score

Variable	ML score (95% CI)^a^	MEWS (95% CI)^b^	Difference (95% CI for difference)^c^	*P *value
Cardiac arrest within 72 hours after presentation				
Area under ROC curve	0.781	0.680	0.101 (0.006 to 0.197)	0.037
Sensitivity	81.4	74.4	7.0 (-11.1 to 21.9)	0.581
Specificity	72.3	54.2	18.1 (14.3 to 22.0)	< 0.001
Positive predictive value	12.5 (9.0 to 17.1)	7.4 (5.3 to 10.3)		< 0.001
Negative predicting value	98.8 (97.5 to 99.4)	97.8 (95.9 to 98.8)		0.133
Likelihood ratio (+)^d^	2.94 (2.46 to 3.52)	1.62 (1.34 to 1.96)		
Death after admission^e^				
Area under ROC curve	0.741	0.693	0.048 (-0.023 to 0.119)	0.185
Sensitivity	69.8	74.4	-4.7 (-16.7 to 7.4)	0.572
Specificity	73.9	55.7	18.2 (14.3 to 22.2)	< 0.001
Positive predictive value	21.5 (16.9 to 26.9)	14.7 (11.5 to 18.4)		< 0.001
Negative predicting value	96.0 (94.1 to 97.3)	95.5 (93.2 to 97.1)		0.608
Likelihood ratio (+)^d^	2.67 (2.23 to 3.20)	1.68 (1.45 to 1.94)		

CI, confidence interval; MEWS, modified early warning score; ML, machine learning; ROC, receiver operating characteristic. ^a^A cutoff value of 60 and above was used for the ML score. ^b^A cutoff value of 3 and above was used for the MEWS. ^c^The 95% confidence interval for the difference between the ML score and the MEWS for each diagnostic statistic was calculated, except for positive predictive value, negative predictive value, and likelihood ratio (+) that are not well established. ^d^Likelihood ratio of a positive test. ^e^In-hospital death during current admission. 95% confidence interval or statistical test not computed because the method is not well established for the diagnostic statistics concerned.

## Discussion

The results of this study showed that a ML score incorporating vital signs and HRV parameters is more predictive of cardiac arrest within 72 hours of presentation to the ED compared with the MEWS. Categorization of patients into low, intermediate, and high risk groups according to the ML score is a useful predictor of risk for cardiac arrest and death. The ML score represents a non-invasive and objective risk-stratification tool that can be determined immediately at presentation to the ED. As diagnosis in the ED setting is often time dependent, medical decisions in the ED (disposition, level of monitoring, aggressive management) are often made based on risk assessment, rather than on diagnosis. We believe this is the first study to show the potential of a ML model incorporating age, vital signs, and HRV for predicting cardiac arrest and death.

Our study also indicates that HRV measured from short-term ECG recordings (5 to 30 minutes), when combined with vital signs, provides a useful tool for risk stratification in the ED. In this study, depressed HRV parameters were associated with early (72 hours) adverse cardiac events and death after admission (Table 3). This is consistent with the findings of previous studies that suggest short-term measurement of frequency-domain HRV parameters is strongly associated with cardiac death and mortality [11-13,32].

Decreased HRV has been found to predict increased mortality in the older patient [33], and for coronary artery disease [14,34], post-myocardial infarction [11], congestive heart failure [35], and dilated cardiomyopathy [36]. Altered spectral HRV analysis has been found to be an indicator of severity in congestive heart failure [37], hypertension [38], coronary artery disease [39], angina [40], myocardial infarction [41], hypovolemia, hypoxia [42], chronic renal failure [43], and diabetes mellitus [44]. Decreased HRV has also been found in ICU patients following head trauma [45-49], sepsis [50], and septic shock [51,52]. HRV has also been used as a marker of severity in ED patients with sepsis [53]. Depressed HRV may reflect a decrease in vagal activity directed to the heart that leads to prevalence of sympathetic mechanisms [54], and therefore to cardiac instability, which might explain the higher risk of arrhythmic deaths [14,55]. However, the true sympatho-vagal correlates of HRV and the mechanisms behind reduced HRV still remain largely unknown [56].

In our previous research, we proposed using a combination of age, HRV measures, and vital signs as a predictor of patient outcomes and demonstrated that the combined features present significant improvements to predictive accuracy, sensitivity, and specificity compared with using HRV alone [22,57]. As we can see from Table 3 not all of the vital signs were highly predictive when used in isolation. HRV parameters also tend to be highly correlated. By using a ML approach, we were able to overcome some of these limitations as well as the overfitting associated with traditional statistical methods [58]. We have investigated an extreme learning machine and a support vector machine with different activation/kernel functions as classifiers, and found that the linear support vector machine is able to provide the highest confidence in categorizing patients into two outcomes: death and survival. Furthermore, we have also presented a new segment-based decision-making strategy for outcome prediction [22].

### Limitations

Several limitations are inherent in our study. This study was carried out in a single-center study at a tertiary teaching hospital in Singapore and the results may not be generalizable to other settings. However, we are confident that the current dataset accurately represents the management of ED patients in our hospital.

The diverse types of conditions in the patients recruited may have different effects on the MEWS and ML scores. The main diagnosis groupings are cardiovascular, respiratory, neurological, gastrointestinal, renal, endocrine, infectious disease, vascular, trauma, cancer, and others. The different diagnosis groupings may cause inaccuracies in predicting the ML score because the HRV in noncardiovascular conditions may differ from that in cardiovascular-related conditions. In future study, therefore, all of the different subgroups should be analyzed separately.

Another limitation of our study is that while the ML score has been shown to have good internal validity, there is a need for external validation of the score for routine clinical use.

One of the exclusion criteria of this study was the exclusion of patients in nonsinus rhythm. It remains to be seen whether the ML score will remain a good predictor of adverse outcomes in patients with irregular heart rhythms. Previous studies in patients with atrial fibrillation have found an association between HRV and an increased risk for cardiac death [59], as well as recurrence of atrial fibrillation [60]. Patients in atrial fibrillation, however, only represent a minority of patients, estimated to be approximately 2 to 4% in patients between ages 60 and 79 [61] and < 1% in patients below the age of 55 [61]. Another limitation of our study is the lack of follow-up for patients discharged from the ED. Electronic medical records of patients that were discharged before 72 hours were also checked for any admissions to public hospitals in Singapore to minimize missed cases of adverse cardiac events.

The ML score is also based on a single ECG recording. Although this represents a rapid method for risk stratification, not much is known about the change of HRV variables over time. Studies have suggested that acute changes in HRV occur before the onset of ventricular tachycardia [62-65]. Changes over time in HRV have also been found to occur in the early phase of recovery after myocardial infarction [66-68]. Serial analyses of changes in HRV were not performed in our study and should be investigated for in follow-up studies.

### Future studies

The results of this study should be validated with a larger sample size, in view of the rare outcome of cardiac arrest within 72 hours or death. Further studies should also be carried out to validate the ML score in a prospective series in patients with different diagnosis groupings.

We have since developed a laptop-based prototype to acquire real-time signals and to process and analyze HRV parameters. This device incorporates ECG and other vital signs such as blood pressure, pulse oximetry, and respiratory rate, together with clinical data, for instantaneous, intelligent prediction of cardiac arrest and mortality using neural networks. In the future, we aim to prospectively validate the prediction scores generated by our device with critically ill patients, including the assessment of the effect of ongoing treatment on our prediction index and survival. Further development is also needed to produce a stand-alone device, ready for clinical use and possible clinical trials. We believe that there exists potential for the development of bedside devices capable of real-time monitoring of HRV, which may help physicians to identify patients at high risk for cardiac arrest and death.

## Conclusion

In critically ill patients presenting to the ED, we found ML scores to be more accurate than the MEWS in predicting cardiac arrest within 72 hours. The results of this study also suggest that initial short-term HRV measurements, in addition to vital signs, may play a role for early, rapid, and objective risk stratification of patients during triage.

## Key messages

• We determined the relationship of the predictor factors with the outcome of cardiac arrest within 72 hours and death after admission. Significant association (*P *< 0.05) with the primary outcome included the GCS, pulse rate, respiratory rate, SpO_2_, aRR, avHR, sdHR, RR triangular index, LS-VLF power, LS-HF power, LS-LF norm, LS-HF norm, and MEWS.

• Those factors found to have significant association (*P *< 0.05) with the secondary outcome included the GCS, respiratory rate, SpO_2_, aRR, avHR, RMSDD, RR triangular index, TINN, LS-VLF power, LS-HF power, LS-LF norm, LS-HF norm, LF/HF ratio, and MEWS.

• The AUROCs of the ML score for both primary and secondary outcomes (0.781 and 0.741, respectively) are higher compared with those for the MEWS (0.680 and 0.693, respectively).

• The sensitivity and specificity of the ML score are 81.4 (95% CI: 66.1 to 91.1) and 72.3 (95% CI: 69.2 to 75.2), respectively; both are higher compared with the sensitivity and specificity of MEWS (74.4, 95% CI: 58.5 to 86.0; and 54.2, 95% CI: 50.8 to 57.5, respectively).

• In critically ill patients presenting to the ED, we found ML scores to be more accurate than the MEWS in predicting cardiac arrest within 72 hours and death.

## Abbreviations

AUROC: area under the receiver operating characteristic curve; AVPU: A for 'alert', V for 'reacting to vocal stimuli', P for 'reacting to pain', U for 'unconscious'; CI: confidence interval; ECG: electrocardiogram; ED: emergency department; GCS: Glasgow Coma Scale; HRV: heart rate variability; MEWS: modified early warning score; ML: machine learning; PACS: Patient Acuity Category Scale; ROC: receiver operating characteristic; SpO_2_: oxygen saturation.

## Competing interests

MEHO and ZL have a patent filing related to the technology described in the study (Method of predicting acute cardiopulmonary events and survivability of a patient; application number 13/047,348). They also have a licensing agreement with ZOLL Medical Corporation for the technology. All remaining authors declare that they have no competing interests.

## Authors' contributions

MEHO planned and established the project, including the procedures for data collection, drafted the manuscript, and performed data analysis. SF-C performed detailed statistical analysis of the data. CHLN drafted the manuscript, and performed data collection and data analysis. KG, NL, ZXK, NS, and TTZ performed data collection and data analysis. ZL reviewed critical revisions to the manuscript. All authors took part in rewriting and approved the final manuscript.
